# Morphological MRI features as prognostic indicators in brain metastases

**DOI:** 10.1186/s40644-024-00753-0

**Published:** 2024-08-20

**Authors:** Beatriz Ocaña-Tienda, Julián Pérez-Beteta, Ana Ortiz de Mendivil, Beatriz Asenjo, David Albillo, Luís A. Pérez-Romasanta, Manuel LLorente, Natalia Carballo, Estanislao Arana, Víctor M. Pérez-García

**Affiliations:** 1 Mathematical Oncology Laboratory (MOLAB), University of Castilla-La Mancha, Ciudad Real, Spain; 2 Sanchinarro University Hospital, HM Hospitales, Madrid, Spain; 3 Hospital Regional Universitario de Málaga, Málaga, Spain; 4 MD Anderson Cancer Center, Madrid, Spain; 5 Salamanca University Hospital, Salamanca, Spain; 6 Fundación Instituto Valenciano de Oncología, Valencia, Spain

**Keywords:** Brain metastasis, MRI, Biomarkers, Radiation therapy, Response, Morphological features

## Abstract

**Background:**

Stereotactic radiotherapy is the preferred treatment for managing patients with fewer than five brain metastases (BMs). However, some lesions recur after irradiation. The purpose of this study was to identify patients who are at a higher risk of failure, which can help in adjusting treatments and preventing recurrence.

**Methods:**

In this retrospective multicenter study, we analyzed the predictive significance of a set of interpretable morphological features derived from contrast-enhanced (CE) T1-weighted MR images as imaging biomarkers using Kaplan–Meier analysis. The feature sets studied included the total and necrotic volumes, the surface regularity and the CE rim width. Additionally, we evaluated other nonmorphological variables and performed multivariate Cox analysis.

**Results:**

A total of 183 lesions in 128 patients were included (median age 61 [31–95], 64 men and 64 women) treated with stereotactic radiotherapy (57% single fraction, 43% fractionated radiotherapy). None of the studied variables measured at diagnosis were found to have prognostic value. However, the total and necrotic volumes and the CE rim width measured at the first follow-up after treatment and the change in volume due to irradiation can be used as imaging biomarkers for recurrence. The optimal classification was achieved by combining the changes in tumor volume before and after treatment with the presence or absence of necrosis (*p* <  < 0.001).

**Conclusion:**

This study demonstrated the prognostic significance of interpretable morphological features extracted from routine clinical MR images following irradiation in brain metastases, offering valuable insights for personalized treatment strategies.

**Supplementary Information:**

The online version contains supplementary material available at 10.1186/s40644-024-00753-0.

## Background

Brain metastases (BMs) are the most common intracranial tumors in adults, the incidence of which is estimated to be 10% to 30% of all oncological patients [1]. The number of BMs detected is increasing due to both the improved detection of small metastases by higher spatial and contrast resolution in medical images and the increase in the number of patients affected by primary cancers and their longer survival [2]. Despite the high incidence of brain metastases, relatively few studies looking for relevant biomarkers have been carried out in this field, in comparison to those on other brain tumors [3], in part due to the lack of large patient datasets [4].

Stereotactic radiotherapy (SRT), delivered as either multiple fractions (FSRT) or as a single session of high-dose treatment (SRS), has become the therapy of choice for the management of BMs. In the current era of immunotherapy and targeted therapies for potentially increased systemic disease survival, 10 or more BMs are routinely treated with SRS alone at most medical centers [4]. However, even after SRT, some tumors do not respond or recur, and this growth may persist in up to 12% of patients after 15 months [5]. At the treatment planning stage, it is unclear which lesions will recur. The development of non-invasive imaging biomarkers might improve patient selection and help in identifying potential non-responders, as patients may need early treatment adjustments or salvage treatments if they are found to be at a high risk of failure.

The diagnostic and prognostic value of quantitative imaging has been extensively demonstrated in numerous studies [6–8]. While several authors have qualitatively identified prognostic factors for BMs [9–11] and others have explored the presence of necrosis in surgically treated BMs [12], the investigations of quantitative prognostic indicators for local control after SRT are limited [13].

Therefore, the primary objective of this study is to evaluate specific geometric features extracted from contrast-enhanced (CE) T1-weighted (T1w) MR images obtained at baseline or within 12 weeks after SRT. These features, such as surface regularity, total volume, or the amount of necrotic tissue within the tumor, will be investigated as potential predictors of recurrence in BMs.

## Methods

### Patients

All the patients included in this study were participants in a retrospective, multicenter, non-randomized study authorized by ethics boards at five institutions. A prior study included patients diagnosed with BM between 2007 and 2021 and followed up with MR scans according to standard clinical practice [14]. The inclusion criteria were as follows: (i) had undergone SRT, fractionated stereotactic radiotherapy or single-session stereotactic radiotherapy at any point during the course of the disease; (ii) had a longest diameter of at least 10 mm before SRT; and (iii) had access to volumetric CE MR images throughout the entire follow-up (slice thickness < 2 mm). Table 1 provides more information about the subjects involved in the study.

**Table 1:** Baseline characteristics of patients in study

	**n (%)**
**Sex**	
Male	64 (50.0)
Female	64 (50.0)
**Age**	
Median (range)	61 (31–95)
**Primary tumor histology**	
NSCLC	75 (58.6)
Breast	26 (20.3)
SCLC	10 (7.8)
Melanoma	2 (1.6)
Others	15 (11.7)
**Number of metastases at diagnosis**	
1	63 (49.2)
2	36 (28.1)
3	16 (12.5)
≥ 4	13 (10.2)
**Graded Prognostic Assessment (GPA)**	
Median (range)	2.5 (1–4)
**Radiation therapy**	
Single-session stereotactic radiosurgery	73 (57.0)
Prescription dose (Gy) [median (range)]	20 (16–24)
Fractionated stereotactic radiotherapy	55 (43.0)
Number of fractions [median (range)]	5 (3–10)
Dose per fraction (Gy) [median (range)]	5.5 (2.5–20)
Previous whole brain radiotherapy (WBRT)	20 (15.6)
**Overall survival (months)**	
Median (range)	12.77 (4.17 – 102.23)

### MR imaging

The volumetric CE-T1w MR imaging sequence used to delineate the BMs and compute their volumes was gradient echo using 3D spoiled gradient-recalled echo or 3D fast-spin echo after intravenous administration of a single dose of gadolinium-based contrast agents (GBCAs) with a 6-to-8-min delay. MRI was performed in the axial or sagittal plane with a 1.0 T (*n* = 7), 1.5 T (*n* = 523) or 3.0 T (*n* = 141) MR imaging unit. The imaging parameters included a slice thickness of 0.5–2.0 mm (median 1.3 mm) and 0.4–1.0 mm (median 0.5 mm) for pixel-spacing.

### Radiation therapy, study endpoints and response assessment

All the BMs in the study received SRT treatment. Thirty lesions had previously undergone whole-brain radiation (WBRT). The median time between the end of WBRT and SRT was 6.5 months (1.3–15.2).

Patients were followed up with a volumetric MRI scan and clinical follow-up appointment every 2–3 months after SRT. All available MR images from pre-SRT until either second radiotherapy or surgery, or a maximum of two years following SRT, were segmented for the post-contrast T1-w sequence.

The time to progression was used to evaluate BMs and was determined using a volumetric criterion as described in a previous paper [15].

### Tumor segmentation

**Fig. 1:**
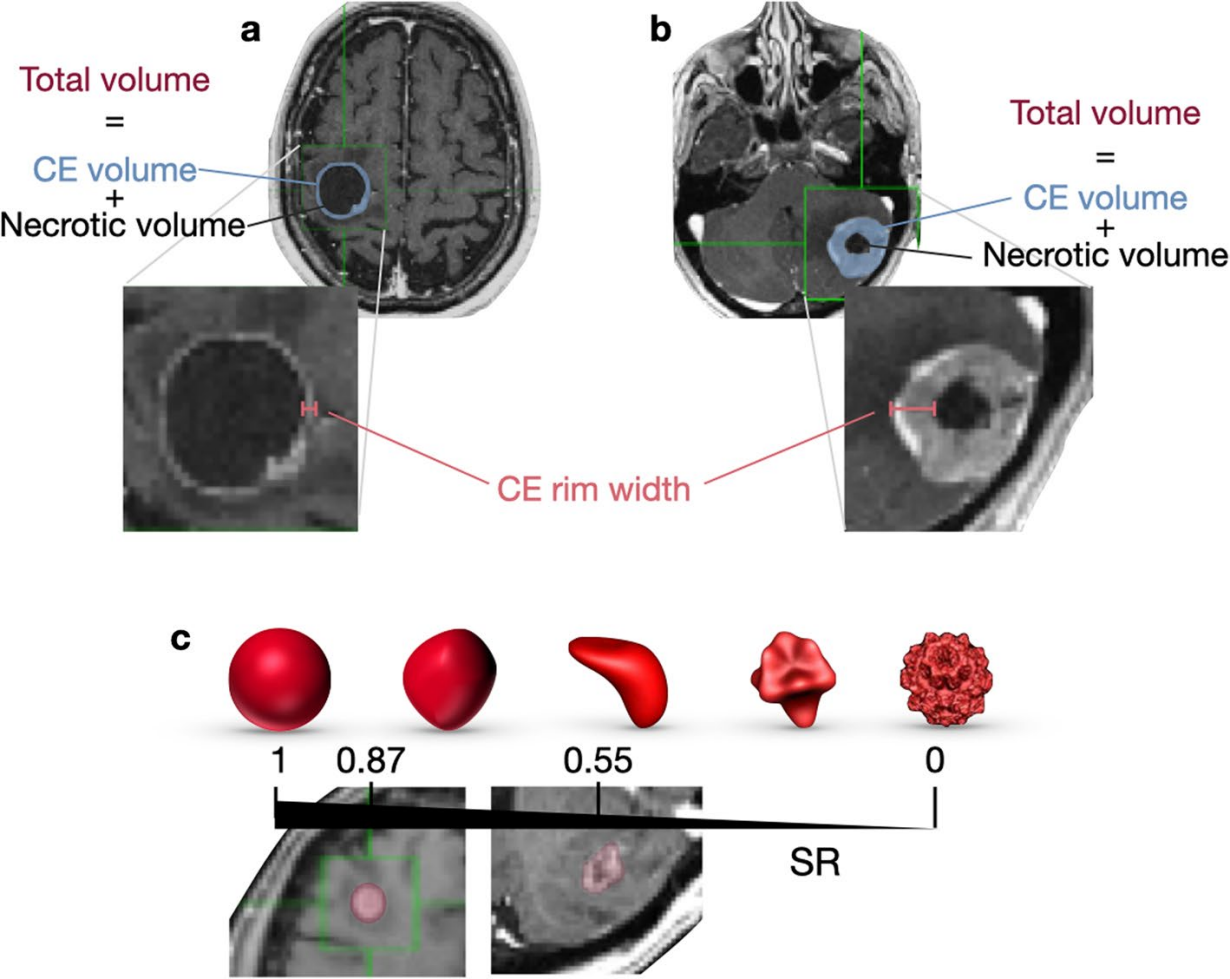
Morphological MRI features described in the study. **a-b** The contrast-enhanced (CE) volume is shown in blue, with the inner black part representing necrosis and both comprising the total volume of the tumor. Additionally, the CE rim width is depicted in red. **c **Surface regularity (SR) ranges from 0 to 1, with 1 representing a perfect sphere. Two examples of BMs are presented, with SR values of 0.87 and 0.55

The retrospective analysis of CE-T1w images was performed by the same imaging expert and reviewed by both an imaging expert with more than six years of experience in tumor segmentation and a senior radiologist with 27 years of experience. The scientific software package MATLAB (R2022b, The MathWorks, Inc., Natick, MA, USA) was used to perform the segmentation by importing the DICOM files. With the help of a gray-level threshold, the CE tumor was selected to automatically define the total tumor volume which is defined as the combined volume of the necrotic and CE components, as depicted in Fig. 1. When needed, segmentation was corrected manually, slice by slice, as described previously [6]. The CE and necrotic areas of the lesions were reconstructed, and the tumor interfaces were rendered in 3D. The tumor was computed as the volume inside the surface boundaries that define CE regions.

### Surface regularity and CE rim width

Two of the quantitative measures examined were surface regularity (SR) and CE rim width. Tumor surface regularity is the relationship between the surface area and volume of the lesion. The surface regularity as described in [6] is defined by.

$$\text{SR}=10.63\frac V{\sqrt{S^3}},$$

where $V$ is the total volume of the tumor and $S$ is its rendered surface area (see Fig. 1c). The CE rim width determines the average width of the CE areas by assuming that the regions of necrotic tissue and the whole tumor are spherical and is defined by

$$\text{CE rim width}=0.62\left(\sqrt[3]{{V}_{T}} - \sqrt[3]{{V}_{n}}\right)$$

where ${V}_{T}$ is the total volume of the tumor and ${V}_{n}$ is the necrotic volume of the lesion (see Fig. 1a-b). Necrosis was defined as central regions with no contrast enhancement and ring-enhancing areas around them.

### Statistical analysis

Time-to-event outcomes were calculated from the SRT onset to MRI volumetric progression date, according to the criteria described above [15]. Progression-free survival events were evaluated using the Kaplan–Meier estimator in MATLAB. *p*-values less than 0.05 were considered to indicate statistical significance. The optimal threshold value was sought for each variable. A sweep was carried out for the variable's threshold between its minimum and maximum values, the sample was split into two different subgroups each time, and the log-rank *p*-value was computed for each. The non-isolated significant value with the lowest log-rank *p*-value was selected as the best [6].

The Wilcoxon signed-rank test, a nonparametric statistical hypothesis test, was used to compare the locations of two populations using two matched samples. A Spearman correlation coefficient greater than 0.7 was considered to indicate a strong correlation and was used to indicate correlations between pairs of variables. The normality of the variables was evaluated by the Kolmogorov–Smirnov test.

We employed multivariate proportional hazard Cox analysis using the stepwise Wald method to develop predictive models. This approach assesses a group of variables and gradually eliminates the variable with the lowest statistical significance. SPSS software (v.25) was used for statistical analysis.

## Results

A total of 672 segmentations/time points were examined for 183 lesions in 128 patients. The average number of follow-ups for each BM was thus 3.6. The BMs of each patient were first identified, the images were processed, and the segmentation was carried out to isolate the region of interest. Then, the tumors were reconstructed, geometrical features were extracted, and finally, using clinical data, each variable was classified and examined to determine whether it could be used as a biomarker.

### Clinical variables

Several clinical variables were examined as possible prognostic factors. One of these variables was the Graded Prognostic Assessment (GPA) score, which is used to stratify disease severity and guide treatment decisions, including enrollment in clinical trials [16]. However, within our cohort, GPA did not correlate with either progression-free survival (PFS) or overall survival, as depicted in Supp. Fig. S1. The only significant prognostic predictor was the total prescription dose to each lesion. This predictor showed no correlation with the total volume of the lesion, regardless of whether the radiation was delivered as a single fraction or multiple fractions, as shown in Supplementary Figure S2. The number of metastases at diagnosis did not show statistically significant differences, as did sex and age. These are described in Table 2.

**Table 2:** Results of univariate Cox and Kaplan–Meier analyses for clinical variables

	**Median difference (months)**	**Best** **threshold**	***p*** value	**HR**
**GPA (PFS)**	4.9	2.5	0.212	0.699 (0.411, 1.190)
**GPA (Overall Survival)**	2.2	2	0.151	0.719 (0.457, 1.131)
**Number of metastases**	-	1	0.71	1.089 (0.693, 1.714)
**Sex**	-	-	0.769	1.077 (0.656, 1.770)
**Age**	5.0	66	0.168	0.676 (0.386, 1.184)
**Prescription dose**	7.1	18.5	*p* < < 0.001	0.395 (0.247,0.633)

*HR* Hazard Ratio, *GPA* Graded Prognostic Assessment, *PFS* Progression free survival. *P* values correspond to the log rank test, and the data in parentheses are 95% confidence intervals for the HR

### Imaging biomarkers obtained from MR images

Five distinct quantitative metrics were assessed based on pre-treatment MR images: total volume, necrotic volume, presence or absence of necrosis, SR, and width of the CE rim. None of these measurements achieved statistical significance when considered potential biomarkers, as detailed in Table 3.

**Table 3:** Results of univariate Cox and Kaplan–Meier analyses of imaging biomarkers obtained from pre-treatment and post-treatment RM images

	**Median difference (months)**	**Best** **threshold**	***p*** value	**HR**
**Pre-treatment**				
**Total Volume (cm**^**3**^**)**	3.3	8.30	0.051	1.009 (0.984, 1.035)
**Necrotic Volume (cm**^**3**^**)**	3.3	0.10	0.097	1.622 (0.909, 2.896)
**Necrosis yes/no**	-	-	0.682	0.896 (0.528, 1.520)
**Surface Regularity**	-	0.64	0.110	6.91 (0.257, 185.64)
**Surface Regularity (> 3 cm**^**3**^**)**	4.9	0.65	0.032	2.384 (1.049, 5.422)
**CE rim width (cm)**	3.6	0.60	0.234	0.759 (0.481, 1.198)
**Post-treatment**				
**Total Volume (cm**^**3**^**)**	8.6	1.17	*p* < < 0.001	3.510 (2.199, 5.603)
**Necrotic Volume (cm**^**3**^**)**	7.3	0.09	0.004	3.039 (1.932, 4.779)
**Necrosis yes/no**	6.4	-	0.021	1.919 (1.090, 3.379)
**Surface Regularity**	5.5	0.65	0.068	1.525 (0.965, 2.408)
**CE rim width (cm)**	5.1	0.49	*p* < < 0.001	2.609 (1.661, 4.098)
**Total Volume (post/pre)**	7.4	0.50	*p* < < 0.001	3.610 (2.268, 5.747)

*HR* Hazard Ratio, *CE* Contrast enhanced. *P* values correspond to the log rank test and data in parenthesis are 95% confidence intervals for the HR

**Fig. 2:**
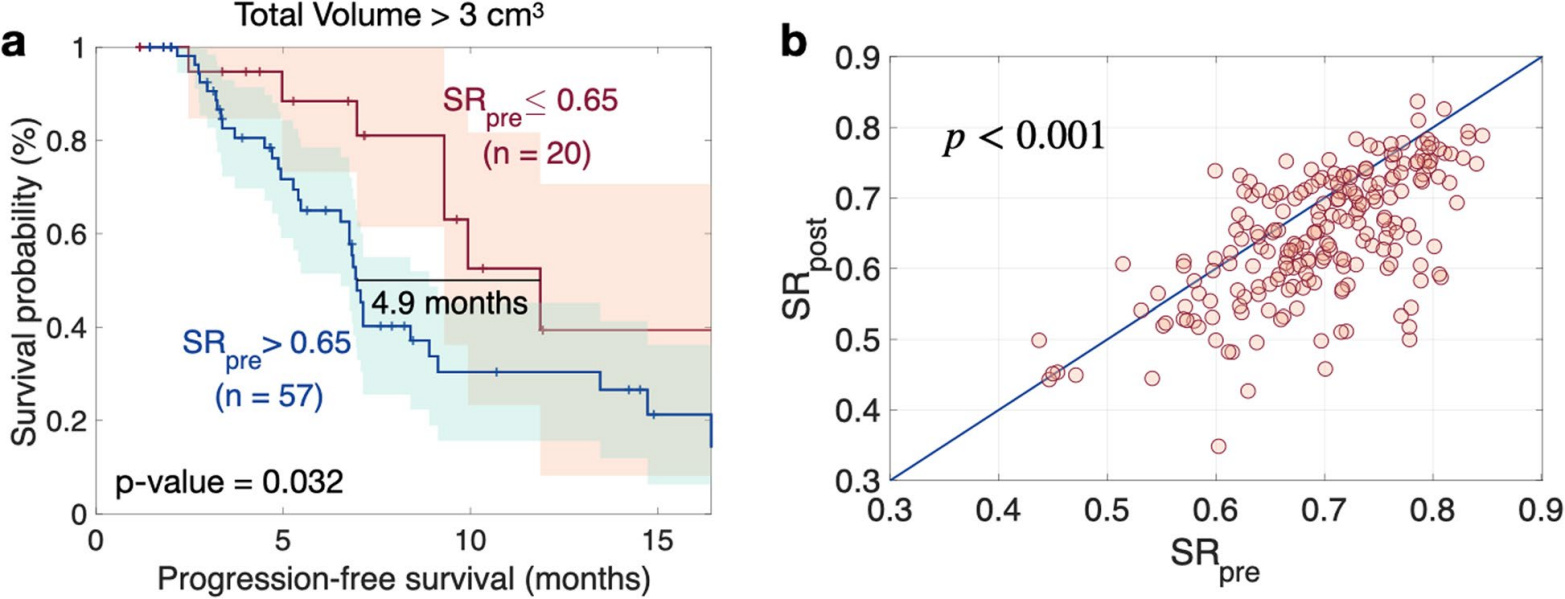
**a** Kaplan–Meier plot per-lesion for the surface regularity (SR) in pre-treatment BMs larger than 3 cm^3^ (*n* = 77). The *p*-value corresponds to the log-rank test. **b** Scatter plot displaying the SR values for BMs before and after treatment (first follow-up, *n* = 183). The *p*-value corresponds to the Wilcoxon signed-rank test

However, for lesions larger than 3 cm^3^, the SR = 0.65 differentiated between two groups with different intervals to progression, with a median difference of 4.9 months (*p* = 0.032). The irregular lesions subgroup (SR ≤ 0.65) had a better prognosis than did those in the more regular lesion subgroup (Fig. 2a). This finding is consistent with the fact that, in our dataset, the SR decreased after radiation treatment (*p* < 0.001), as depicted in Fig. 2b.

At the first post-treatment MRI scan, conducted approximately 3 months after treatment, we further examined the same five measurements. We also evaluated the ratio of total volume between post- and pre-treatment measurements. All the variables, except for the SR, demonstrated predictive value, as described in Table 3 (Suppl. Fig S3).

A statistically significant and robust threshold was identified for the total volume, defined as the sum of the CE volume and the necrotic volume. BMs with post-treatment volumes under 1.2 cm^3^ exhibited longer PFS. Furthermore, when considering the necrotic volume, BMs with less than 0.1 cm^3^ demonstrated a significantly extended PFS, with a median difference of 7.3 months (*p* = 0.004).

Regarding the presence of necrosis, the two subgroups exhibited a PFS difference of 6.4 months, where lesions lacking necrosis had a more favorable prognosis (*p* = 0.021). Although the SR of post-treatment BMs was not a significant prognostic factor (*p* = 0.068), a notable trend persisted, mirroring the findings observed in pre-treatment BMs. Comparisons of the CE rim width between BMs revealed an improved prognosis for lesions with a wider rim (> 0.49 cm). Additionally, BMs whose volumes at the first follow-up after SRT were less than half the baseline volume were associated with prolonged PFS, with a statistically significant *p*-value of < 0.001 and a median difference of 7.4 months.

When the BMs treated with both WBRT and SRT (*n* = *30*) were excluded, the results were consistent with those observed for the full dataset. The same measures previously identified as effective biomarkers of recurrence showed statistically significant differences between the subgroups, as detailed in Supplementary Figure S4.

No differences were observed in the pretreatment volumes between the various types and subtypes of primaries (Figure S5a). However, the SR values for the entire set of BMs from the breast were found to be lower than those for the remainder of the primaries, while melanomas were, in general, larger than the others (Figure S5b). Upon examination of the various subtypes of breast cancer, triple-negative breast cancers (TNBC) exhibited the largest median values for SR. The CE rim width was found to be similar for all primary types and subtypes (Fig. S5c).

### Multivariate analysis

**Fig. 3:**
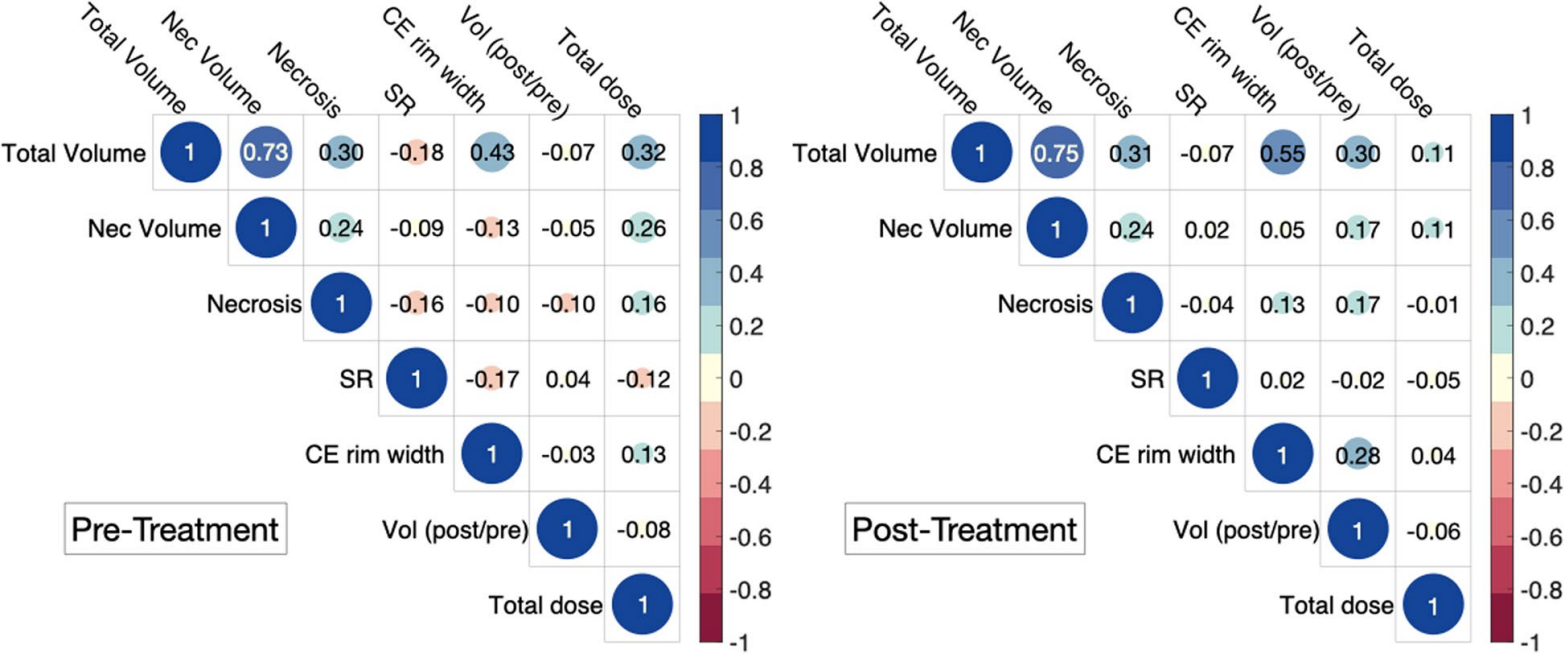
Spearman correlation coefficients between relevant variables of the study. Values larger than 0.7 were regarded as strongly correlated

We investigated combinations of variables to identify an improved predictor. A correlation analysis, illustrated in Fig. 3, demonstrated a significant correlation between total and necrotic volumes, while no significant correlations were observed among the remaining variables. Comprehensive results for all attempted combinations are presented in Supplementary Table S1.

**Fig. 4:**
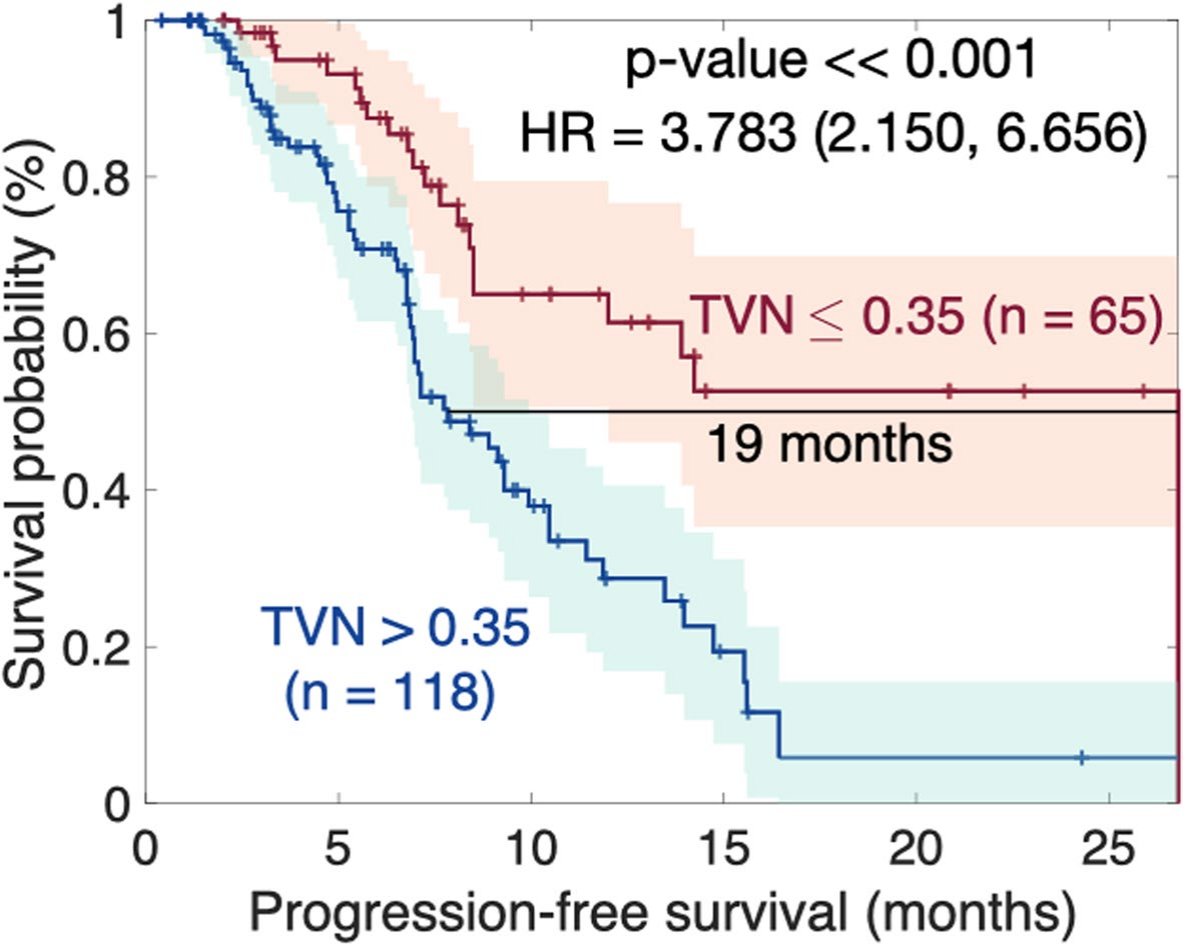
Kaplan–Meier plot per lesion for the multivariate analysis combining the ratio of total volume between post and pre-treatment measurements with the presence or absence of necrosis at the first follow up after treatment. The *p*-value corresponds to the log-rank test. HR – Hazard* Ratio*

The most favorable outcome was achieved when considering the ratio of total volume between post and pre-treatment measurements in conjunction with the presence or absence of necrosis at the first follow-up after treatment,

$${\text{TVN}}=0.084\cdot \text{Total Volume (post/pre)}+0.339\cdot \text{Necrosis (yes/no),}$$

with the median distance between the curves of 19 months (*p* <  < 0.0.1), as depicted in Fig. 4.

## Discussion

This study illustrated that geometric features derived from CE-T1w MR images at the first follow-up, approximately three months post-treatment, can serve as predictive indicators for recurrence in BMs following SRT. These geometric features included total volume, necrotic volume, the presence or absence of necrosis, the CE rim width, and the ratio between volumes before and after irradiation.

We found that radiation therapy made the surface of BMs more irregular, irrespective of their size. For lesions larger than 3 cm^3^, surface regularity on pre-treatment MR images, was found to be a prognostic factor. However, unlike those for other cancers, our finding for BMs was that regular lesions had a worse prognosis. It is well known that more irregular primary melanoma lesions are associated with a worse prognosis [17]. Similar results have been found for other kinds of brain tumors, such as glioblastoma (GB) [6] and meningioma [18]. The same occurs for prostate cancer [19] and lung cancer nodules [20, 21]. It is surprising that the more regular the BMs are, the worse the prognosis is. This finding is consistent with our earlier finding that SRT transforms BMs into more irregular lesions and that it is assumed that SRT also enhances patient prognosis. It would be interesting to study whether the same property holds true for other organ metastases. Understanding why this happens mechanistically requires further investigation. Perhaps irregular primary tumors, which live in their host tissue, are indicators of a mesenchymal phenotype, while metastatic tumors can be effectively screened from the non-host immune system by adopting more compact shapes. This is a very interesting topic that deserves further investigation.

One limitation of the study is that surface regularity is an effective prognostic biomarker only for large lesions. However, those lesions are typically associated with a poorer response to radiation therapy. Consequently, we have developed a method for anticipating the response to treatment in those lesions with a poorer prognosis.

While our study did not reveal statistically significant differences (*p* = 0.051), large BMs at baseline tended to be associated with a poorer prognosis. Notably, an examination of total volume at the first follow-up after radiation treatment suggested that the total volume could serve as a potential prognostic factor, as could the ratio between volumes before and after irradiation. Notably, previous studies have reported no significant association between tumor volume change at 6 or 12 weeks post-SRT and overall survival [22]. However, these studies utilized a 1–3 mm slice thickness in MR images, whereas our dataset exclusively consisted of slices less than 2 mm in thickness. In addition, the use of overall survival to evaluate BMs may be inappropriate because metastatic patients may die from a variety of causes, including systemic disease, intracranial progression, or a combination of both. It has been proven that, in the context of BM patients, overall survival is influenced not only by intracranial control [23] but also strongly by the status of extracranial disease [24, 25].

Previous studies on the predictive/prognostic value of necrotic volume in BMs were purely qualitative, taking into account either the presence or absence of necrosis [10], or semiquantitative among three categories: absence of necrosis, less than 50%, and more than 50% [11]. Here, the qualitative study was repeated by dividing the BMs in our cohort into subgroups with and without necrotic tissue. The current results agree with those of earlier works [26, 27]. However, a quantitative analysis was also carried out, which allowed for an improved distinction between subgroups. The amount of necrotic volume after treatment was a better predictor than the presence or absence of necrosis following SRT. A necrotic volume less than 0.1 cm^3^ is a predictor of good response.

The CE rim width was assessed in previous studies on the morphological features of GBM to assess the relationship between total and necrotic volumes [6]. An analysis of the CE rim width revealed that in the case of BMs, the broader the rim was, the better the prognosis was. These findings contrast with what has been previously reported for GB [6], as happens with the surface regularly, where more regular tumors are linked to a better prognosis.

Previous radiomic studies have found predictor variables such as age and CE-T1w-based kurtosis [28] or an improvement in the classification when features such as the number of metastases, primary tumor site or sphericity are added to the clinical variables [29]. Other studies have used hundreds of features that are not easy to interpret [30, 31] and are susceptible to overfitting, among other issues [32]. A recent study [33] showed that employing two different platforms to extract radiomic features from the same images resulted in inconsistencies and contradicting conclusions, possible because that most radiomic features are not robust [34].

A recent study developed a method to classify post-SRS lesions as either progressive or non-progressive [35]. The authors used data from two centers (*n* = 123 and *n* = 117) and used the maximum diameter in 3 perpendicular directions to evaluate the total volume, with an increase of more than 25% indicating progression. The best classification achieved an AUC = 0.80. Compared to our study, we used data from five institutions and employed similar progression criteria. However, we also took into account the time to progression.

The most innovative part of our study relied on morphological measurements obtained from standard CE-T1w MRI. The computation of such variables can be seen as a time-consuming process. However, volumetric evaluation of BMs has been shown to substantially improve the assessment of BM response to treatments compared with one-dimensional measurements [15], which may suggest the need to incorporate those metrics into clinical practice. The continuous improvement of AI-based tools enabled by the increased availability of BM datasets [36–38] will likely lead to reliable fully automatic segmentation tools in the near future, thus accelerating the process.

Differentiating local recurrent BMs from radiation-induced changes after SRS using contrast-enhanced MRI can be challenging. Approximately one-third of lesions exhibit a transient size increase post-treatment, beginning as early as six weeks and lasting up to 15 months [5]. Between 30 and 75% of SRS-treated BMs that show imaging enlargement are due to radiation-related changes alone. Current structural MRI, relying on contrast enhancement patterns and T2/ fluid-attenuated inversion recovery (FLAIR) alterations, is inadequate for distinguishing tumor recurrence from SRS-induced changes [39]. Post-treatment imaging findings are typically absent, but around 7% of patients may show progression of abnormal hyperintensity on T2-weighted and FLAIR sequences, likely representing edema from increased capillary permeability [40]. In the acute post-treatment setting, increased peripheral enhancement and worsening surrounding vasogenic edema are often due to acute tumoritis and cerebritis, commonly managed with a steroid taper [41]. Although contrast-enhanced T1/T2 mismatch was hypothesized as a useful sign, it has proven ineffective in differentiating radiation necrosis from local recurrence [42]. New leptomeningeal lesions outside the prescription isodose lines should be suspected of progressive disease.

This study has several strengths, the first of which is the careful lesion segmentation process. The same expert conducted each segmentation semi-automatically, and all the results were verified by a radiologist. Another strength was the multicenter approach of the study, which included lesions from five different institutions. Only morphological features with straightforward interpretations directly obtained from segmentation were used. A recent review on machine learning imaging biomarkers in neuro-oncology [43] concluded that these techniques do not yet generally outperform conventional statistical techniques. This review emphasized the need for larger datasets to facilitate a more comprehensive evaluation.

Our study had a number of limitations. To describe the characteristics of the tumor, only CE-T1w MR images were used. Future research may incorporate additional imaging sequences. Furthermore, due to the retrospective nature of the study, the data were not collected in a predesigned way, and in some cases, significant data were missing. For instance, there was a significant lack of molecular markers beyond the specific tumor histology or adjuvant/concurrent immunotherapy or other therapies. Finally, the study was conducted by assessing each BM; future studies may take into consideration patient-by-patient assessments while accounting for all of their BMs.

## Conclusions

This study revealed the predictive value of relevant morphological imaging characteristics extracted from volumetric CE-T1w MR images of patients with BMs before and after stereotactic radiation therapy. Total and necrotic volumes, the CE rim width and the change in volume in response to treatment were significant independent parameters in terms of the time to progression. However, the best classification was found when using the ratio of total volume between post- and pre-treatment measurements together with the presence or absence of necrosis at the first follow-up after treatment.

### Supplementary Information

Supplementary Material 1. 

## Data Availability

The datasets used and/or analyzed during the current study are available from the corresponding author on reasonable request.

## Abbreviations

BM: Brain metastases
CE: Contrast enhanced
FSRT: Multiple-fraction stereotactic radiotherapy
GB: Glioblastoma
GPA: Graded Prognostic Assessment
PFS: Progression-free survival
SR: Surface regularity
SRT: Stereotactic radiotherapy
SRS: Single session stereotactic radiotherapy
WBRT: Whole-brain radiation
