# Supplementary material for: Morphological MRI features as prognostic indicators in brain metastases
Source: Cancer Imaging. 2024 Aug 20;24:111. doi: 10.1186/s40644-024-00753-0 (PMC11334491; doi:10.1186/s40644-024-00753-0)
Supplement: Supplementary file 1 — Supplementary Material 1. [file 40644_2024_753_MOESM1_ESM.pdf]

Supplementary Information for "Morphological MRI Features as Prognostic Indicators in Brain Metastases"

Beatriz Ocaña-Tienda, Julián Pérez-Beteta, Ana Ortiz de Mendivil, Beatriz Asenjo, David Albillo, Luís A. Pérez-Romasanta, Manuel LLorente, Natalia Carballo, Estanislao Arana, Víctor M. Pérez-García

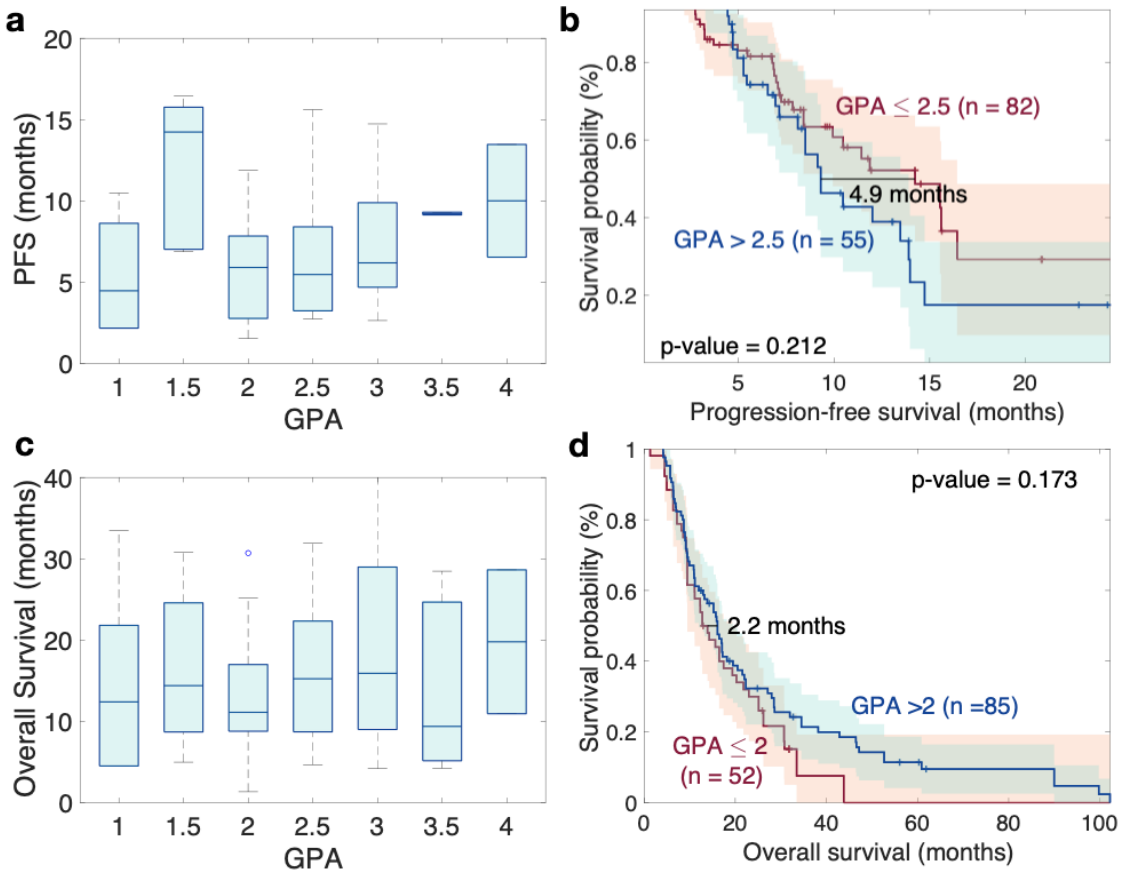

***Figure S1**. Box plots and Kaplan-Meier curves per-lesion illustrating the relationship between the Graded Prognostic Assessment (GPA) and its association with **a-b.** progression-free survival (PFS) and **c-d**. overall survival (n=137).*

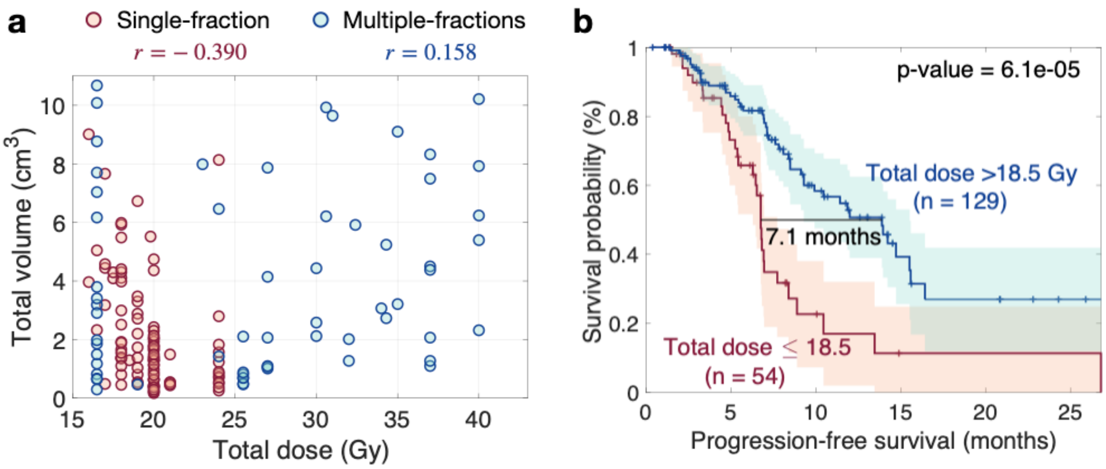

Figure S2. ***a***. *Scattering plot for the total volume and the total received dose in both the case of single fraction and multiple fraction radiation therapies. r-values correspond to the Spearman correlation coefficient.* ***b.*** *Kaplan-Meier curve per-lesion for comparing BMs according to the total received dose.*

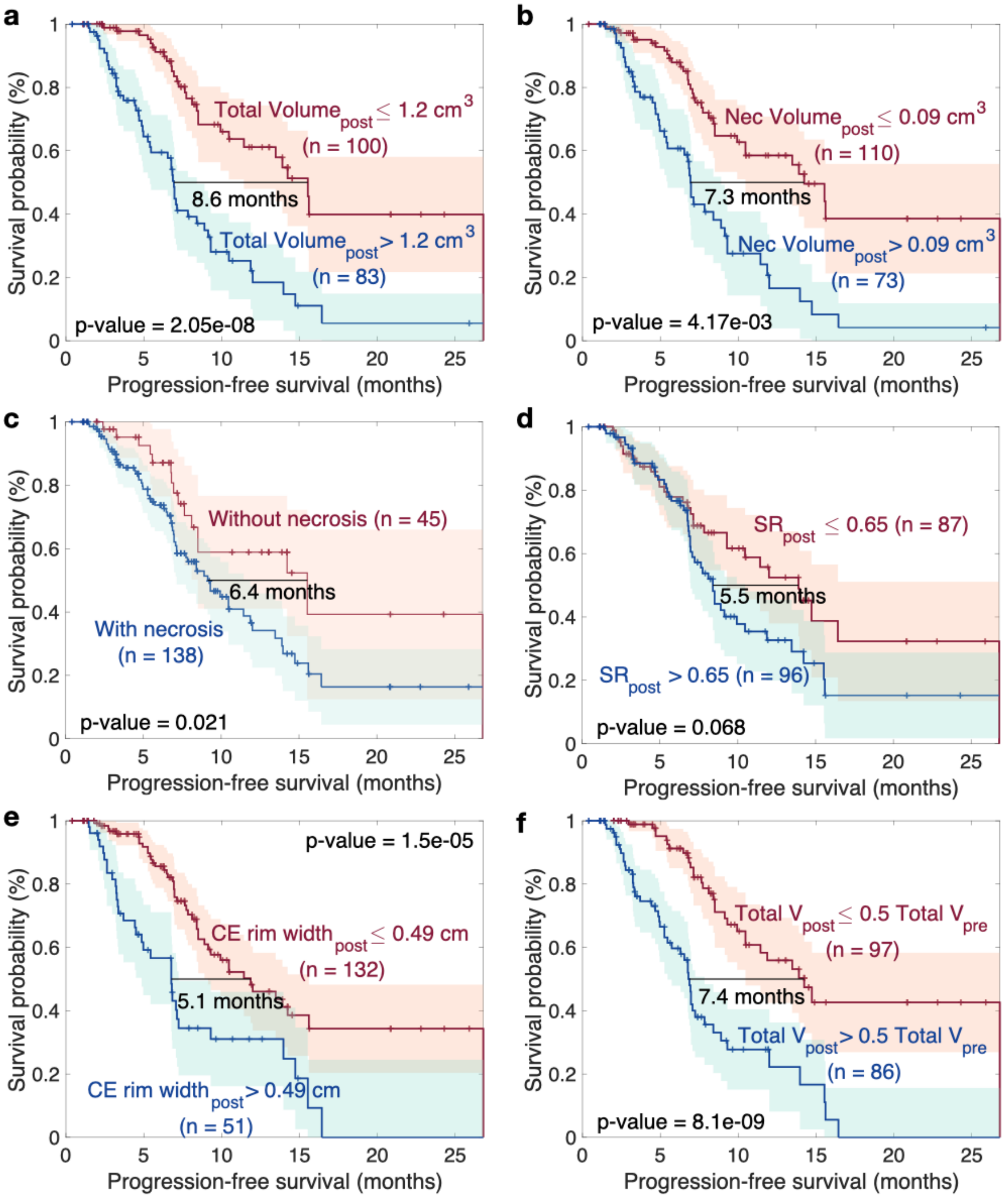

***Figure S3**. **Kaplan-Meier curves per-lesion for biomarkers measured in the first follow-up after treatment, around three months post-treatment.** **a**. Total volume**. b**. Necrotic volume. **c.** Presence of absence of necrosis. **d**. Surface regularity (SR). **e**. CE rim width and **f**. The variations in volume with treatment (Total volume after treatment / total volume before treatment). n=183, p-values correspond to the long-rank test.*

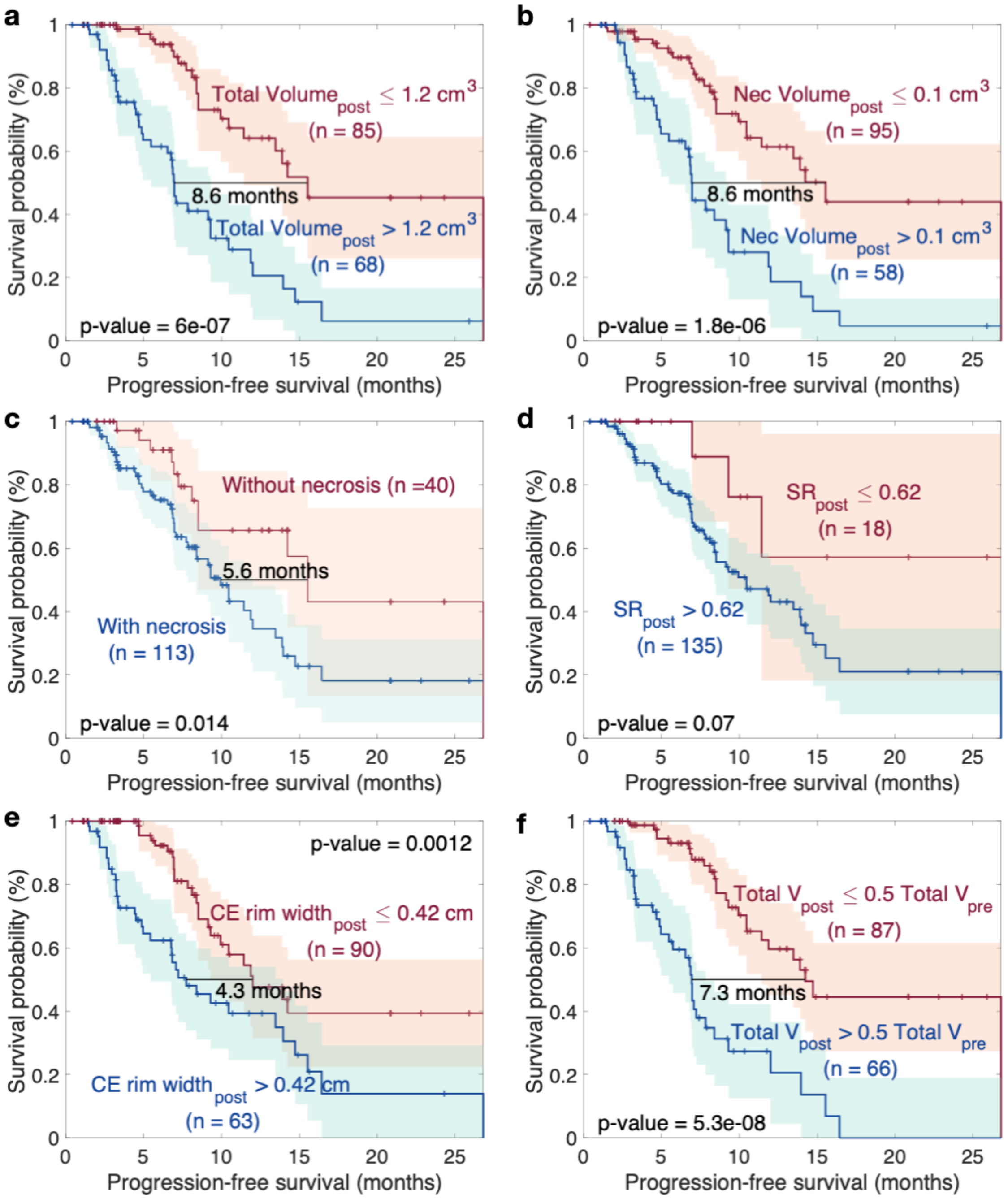

Figure S4. Kaplan-Meier curves per-lesion for biomarkers measured in the first follow-up after treatment, around three months post-treatment, when excluding those previously treated with whole brain radiotherapy (WBRT). a*. Total volume****. b****. Necrotic volume.* ***c.*** *Presence of absence of necrosis.* ***d****. Surface regularity (SR).* ***e****. CE rim width and* ***f****. The variations in volume with treatment (Total volume after treatment / total volume before treatment). n=153, p-values correspond to the long-rank test.*

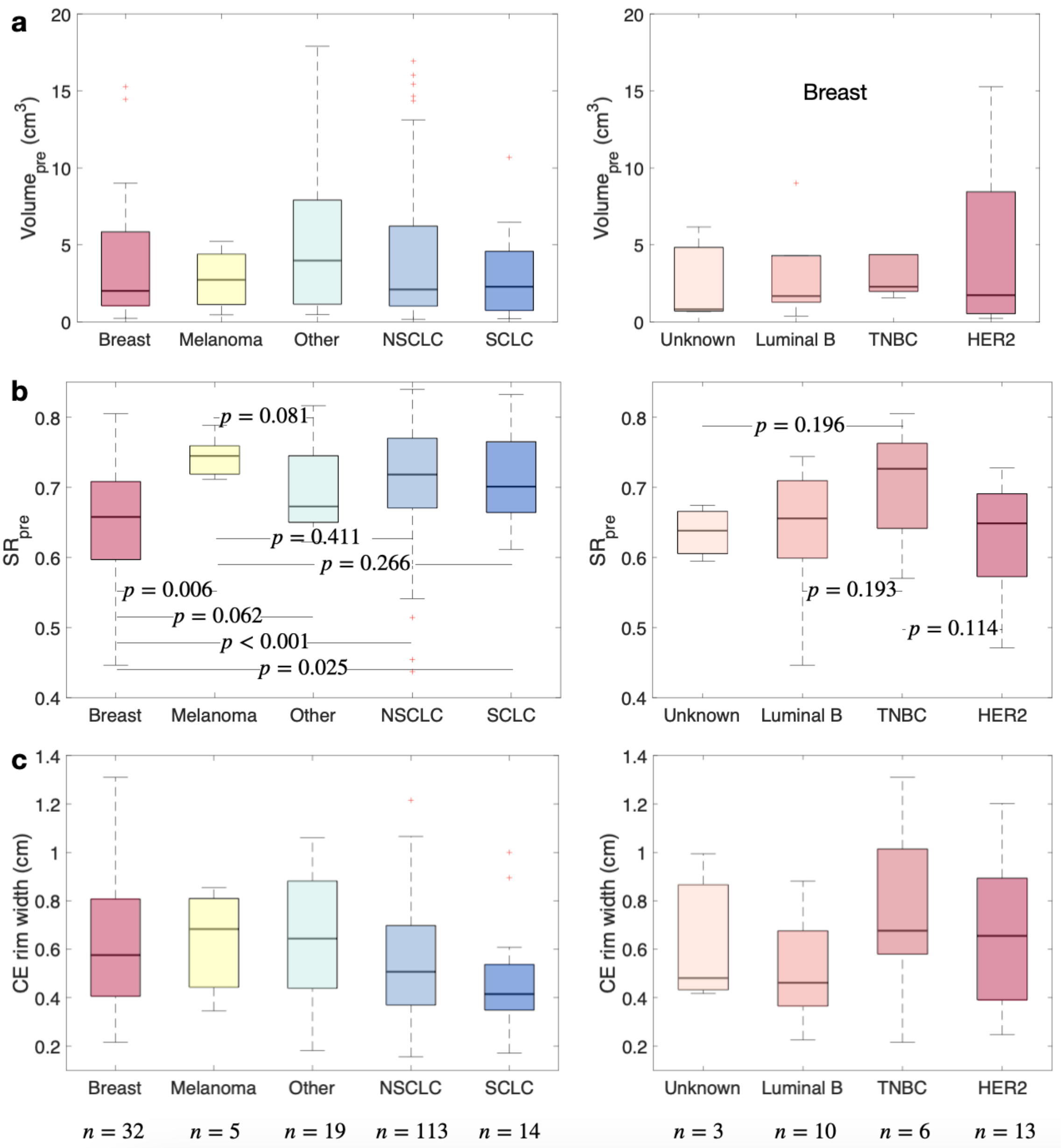

***Figure S5**. **Box plots comparing the values of different morphological variables on pretreatment MRIs for different primary types (left panels) and breast subtypes (right panels)**. **a**. Total volume. **b**. Surface regularity (SR) **c**. Contrast-enhanced (CE) rim width.*

	Median difference (months)	Best threshold	p value	HR
**Total Volume (pre) + total dose**	7.1	-0.9165	3.94E-04	2.283 (1.427, 3.654)
**Total Volume (pre) + SR (pre)**	-	1.90	0.014	2,729 (1.180, 6,312)
**Total Volume (pre) + CE rim width (pre)**	-	0.0381	0.092	1.542 (0.927, 2.566)
**Total Volume (post) + total dose**	7.5	-1.0826	5.49E-11	4.215 (2.647, 6.712)
**Total Volume (post) + necrosis (yes/no, pre)**	6.7	0.1786	1.00E-06	2.938 (1.856, 4,652)
**Total Volume (post) + necrosis (yes/no, post)**	8.6	0.430	1,30E-08	3.571 (2.236, 5.703)
**Total Volume (post) + CE rim width (post)**	7	0.3935	2.40E-05	2.577 (1.631, 4.072)
**Total Volume (post/pre) + total dose**	7.7	-0.3827	1.68E-10	4.028 (2.543, 6.380)
**Total Volume (post/pre) + necrosis (yes/no, pre)**	8.6	0.80	2.80E-11	4.094 (2.612, 6.416)
**Total Volume (post/pre) +necrosis (yes/no, post)**	19.0	0.35	8.56E-07	3.783 (2.150, 6.656)
**Total Volume (post/pre) + CE rim width (post**	7.1	0.919	3.52E-08	3.274 (2.093, 5.122)

Table S1. Results of multivariate Cox and Kaplan-Meier Analysis for imaging biomarkers obtained from pre-treatment and post-treatment RM images. *HR – Hazard Ratio, CE – contrast enhanced. P values correspond to the log rank test and data in parenthesis are 95% confidence intervals for the HR.*
